# Does the Location of Shoe Upper Support on Basketball Shoes Influence Ground Reaction Force and Ankle Mechanics during Cutting Maneuvers?

**DOI:** 10.3390/biology11050743

**Published:** 2022-05-13

**Authors:** Yu Liu, Wing-Kai Lam, Ieva Seglina, Charlotte Apps

**Affiliations:** 1 School of Leisure Sports, Chengdu Sport University, Sichuan 610041, China; liuyu@cdsu.edu.cn; 2 Sports Information and External Affairs Centre, Hong Kong Sports Institute, Sha Tin, Hong Kong, China; 3 School of Science and Technology, Nottingham Trent University, Nottingham NG1 4FQ, UK; ieva.seglina@gmail.com (I.S.); charlotte.apps@ntu.ac.uk (C.A.)

**Keywords:** footwear, joint torque, rotational moment, turn mechanics

## Abstract

**Simple Summary:**

Currently, there are various shoe lateral upper support designs (forefoot, rearfoot, forefoot-to-rearfoot) available in the market, but scientific guidelines are not well established. This study examined the location effect of lateral shoe upper supports on the ground reaction forces, as well as ankle kinematics and moments during the change of direction maneuvers using a statistical parametric mapping approach. The forefoot support shoe had a reduced eversion moment in between ~25–95% across all change of directions. The forefoot upper support shoe had increased ankle inversion between ~8–14% (complete turns) and ~96–100% of contact time (side-cuts and lateral shuffles), and increased inversion velocity in side-cuts. The rearfoot upper support shoes reduced inversion velocity in lateral shuffle compared to no support. These findings suggest that lateral upper support location on basketball shoes can influence coronal plane ankle mechanics, but not ground reaction forces.

**Abstract:**

This study examined the location effect of lateral shoe upper supports on the ground reaction forces, as well as ankle kinematics and moments during the change of direction maneuvers using a statistical parametric mapping approach. University basketball athletes performed side-cuts, complete turns and lateral shuffle maneuvers with their maximum-effort in four shoe conditions with varying shoe upper support locations: full-length, forefoot, rearfoot, none (control). The statistical parametric mapping repeated measures ANOVA test was applied to compare differences between the shoe conditions, followed-up with post-hoc statistical parametric mapping paired *t*-tests between all shoe conditions. The coronal ankle results revealed that the forefoot support shoe had a reduced eversion moment that varied between ~25–95% across all change of directions (*p* < 0.05). However, the forefoot upper shoe had increased ankle inversion between ~8–14% (complete turns) and ~96–100% (side-cuts and lateral shuffles), and increased inversion velocity in side-cuts than the other shoes (*p* < 0.05). Compared to the control, the rearfoot support shoes reduced inversion velocity in side-cut between ~78–92% (*p* < 0.05). These findings suggest that a forefoot upper support induced most changes in ankle mechanics during basketball cutting maneuvers, with only inversion angle in the complete turn being influenced during the initial period where ankle injury may occur. Future research should examine if these coronal ankle mechanics influence change-of-direction performance and injury risk with regular wear.

## 1. Introduction

Acceleration, cutting and turning are increasingly executed in basketball, soccer and other team sports [1,2], as players are adapting with higher level of fitness in modern competition intensity [3]. A game analysis revealed that basketball players change direction (e.g., sidestep cuts, lateral shuffling, turns) every three seconds in a game [4]. These rapid and strenuous whole-body direction changes generate high horizontal ground reaction forces (GRF) and plantar pressures, which may result in excessive joint loading on musculoskeletal structures of the lower extremities [5,6]. The excessive shear and plantar loading are associated with talon noir [7], ankle sprains [8] and non-contact anterior cruciate ligament (ACL) injuries [9].

Previous studies have identified that type/design of footwear could be related to ankle sprain, ACL injury and foot pain during sports [8,10,11]. Appropriate basketball shoes should minimize injury risk potential and promote athletic performances [10]. For instance, higher shoe collar height and greater shear cushioning and outsole traction can improve ankle stability and impact the attenuation and performance of cutting tasks, respectively [6,11,12,13]. In running, neutral shoes and stability shoes have additional inner or external reinforcements that support runners with overpronated feet, reducing ankle inversion during the rolling phase [14]. In basketball, athletic shoes are designed to provide some ankle support by optimizing shoe sole design (e.g., footbed contour, midsole density and extended outsole width) and upper design (e.g., heel counter, collar height, shoe lacing/closure and lateral shoe support) [15,16].

To date, most basketball footwear research has focused on the midsole, whereas the construction of the upper support has received little attention. Some recent studies investigated the effect of shoe fit configurations (lacing or wrap structure) on performance and joint biomechanics in four agility-based movements, including lateral skater jump, countermovement jump, triangle drop step drill and anterior-posterior drill [17,18]. Their results implicated that the shoe lacing/wrap configurations can optimize shoe fit, which improves lower limb alignment and may lower musculoskeletal injury risk [17] and enhance athletic performance [18]. Subramanium and colleagues [19] reported a reinforced upper shoe condition that enabled athletes to perform the lateral shuffles with reduced positive and negative ankle work, which may reduce energy consumption and performances. However, these studies examined the shoe upper effects using only running or training shoes. It should be noted that basketball movements have clear differences in footwear constructions/features and demands compared with running/training shoes [2,20]. This questions the ecological validity of their findings, since players typically wear basketball/court sport footwear when performing maximum lateral movements or jumps. 

Furthermore, the increased frequency and intensity of lateral movements can lead to instability of the forefoot/midfoot regions of shoe upper in basketball shoes [16]. While the vamp (forefoot) and the quarter panels (midfoot) are often made of one piece of material, these upper materials may induce large deformation toward the lateral side relative to the shoe sole while performing lateral movements, which may lead to poorer foot and ankle proprioception [17,21] and higher energy cost [19]. To give added strength to the shoe upper-sole interface, many court shoes are constructed with extended outsoles or midsoles which extend up on the lateral sides to the bottom edge of the upper or have added materials, such as leather/thermoplastic polyurethane to the lateral aspect of the upper shoe [13,16]. To date, there are various lateral upper shoe designs (forefoot, rearfoot, forefoot-to-rearfoot) available in the market, but scientific guidelines are not well established. There appears to be an optimal amount of lateral support to prevent an inversion injury, but enough motion to limit subsequent joint loading. Furthermore, harder materials may increase pressure between the upper shoe and the foot that would affect shoe comfort perception [22] and induce excessive joint loading [23,24], and thereby, athletic performances [25]. Thus, the location of lateral upper support can have implications for injury prevention and enhanced performance in dynamic lateral cutting movements. 

Hence, the purpose of this study was to examine the effects of the lateral shoe upper support location on the ground reaction forces, as well as ankle kinematics and moments in basketball-specific cutting maneuvers (side-cut, complete turn and lateral shuffle). It was hypothesized that lateral shoe upper support would increase ankle stability as indicated by a reduced ankle inversion angle and inversion velocity during basketball-specific cutting maneuvers. It was also hypothesized that an upper support across the full lateral shoe length would increase the ankle moments. 

## 2. Materials and Methods

### 2.1. Participants

A priori power calculation was calculated in G-Power software with an alpha of 0.05 and power of 0.8. Based on the previously reported large effect size between a 45° side-cut and complete turn observed in the peak lower-limb moments and contact time [26], we inputted a large partial eta effect and estimated that 12 participants were adequate for this study. This is similar to other biomechanics studies that compared types of cutting maneuvers (e.g., n = 11 males [27]; n = 13 males [28]; n = 13 males (26)). Therefore, twelve male university basketball athletes (mean ± SD: age 23.3 ± 2.0 y; height 1.78 ± 0.04 m; body mass 67.5 ± 5.9 kg; competition experience 5.1 ± 1.0 y with exposure of 2.7 ± 1.3 times per week) with a shoe size of US 9.0 ± 0.5 were recruited for this study. The shoe size of each participant was confirmed with a foot-length measurement device (Brannock Device, Syracuse, NY, USA). All participants were right-leg dominant, as determined by asking to kick a ball toward a forward target [29]. They reported no lower-limb injuries in the six months prior to data collection. The Research Ethics Committee has approved the study and written consent was obtained by each of the participants. 

### 2.2. Footwear Conditions

**Figure 1:**
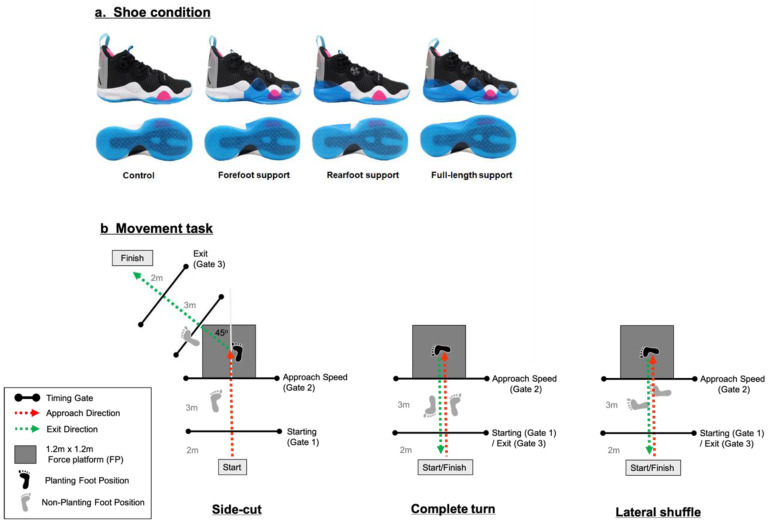
(**a**) Experimental shoe conditions and (**b**) movement courses for side-cut, complete turn and lateral shuffle.

Four identical pairs of basketball shoes (Figure 1a, size US 9.0 Wade 8.0, Li Ning (China) Sports Goods Co., Ltd., Beijing, China), which only differed in the length of lateral upper support [(full-length, forefoot, rearfoot and control) were custom-made for this study. The full-length support shoe had a 1.5 mm thermoplastic polyurethane (TPU) cover laterally from the heel to forefoot (~0–98% of the shoe length, Figure 1a), which was suggested to provide reinforcements to the lateral aspect of the shoe upper [15,16]. The full-length support shoe was identical to the commercial model and commonly used in both professional and amateur players. The forefoot support shoe was built by removing the rearfoot section from the original full-length support (~40–98% of the shoe length, Figure 1a—Forefoot), while the rearfoot support shoe was built by removing the forefoot section from the original full-length support (~0–40% of the shoe length, Figure 1a—Rearfoot). The control had the lateral support completely removed (Figure 1a—Control).

### 2.3. Apparatus and Tasks

To replicate a realistic shoe–ground interface, a 1.2 × 1.2 m force platform (AMTI, Watertown, NY, USA) and its surrounding ground was covered with the standard basketball court surface. Participants were instructed to perform the side-cut, complete turn and lateral shuffle with their maximum effort (Figure 1b), which are commonly investigated in previous studies and basketball training to represent a slight cut, complete direction change and sharp basketball-specific maneuvers, respectively [5,10,11,30,31,32].

For all tested maneuvers, the starting point was set five meters away from the force platform. Photoelectric timing gates (Fusion Sport, Brisbane, Australia) were placed three meters before and after the force platform to measure performance time and to encourage maximum cut velocity. The ellipse time between the starting and exit timing gates indicated the overall cutting performance time across maneuvers. For the side-cut, participants were instructed to sprint forward, plant their right foot on the platform and continue sprinting towards the endpoints at 45 degrees (Figure 1b). For the complete turn, participants were instructed to sprint forward, plant their right foot on the platform and continue sprinting towards the endpoints at 180 degrees (Figure 1b). For the lateral shuffle, participants were asked to shuffle laterally towards the force platform, plant their right foot on the force platform and shuffle back to the starting position [5,31,33]. The force platform (1000 Hz; AMTI, Watertown, NY, USA) and eight-camera motion analysis system (200 Hz; Vicon, Oxford Metrics Ltd., Oxford, UK) were synchronized to record the ground reaction forces and 3D kinematics during each cutting trial.

### 2.4. Procedures

**Figure 2:**
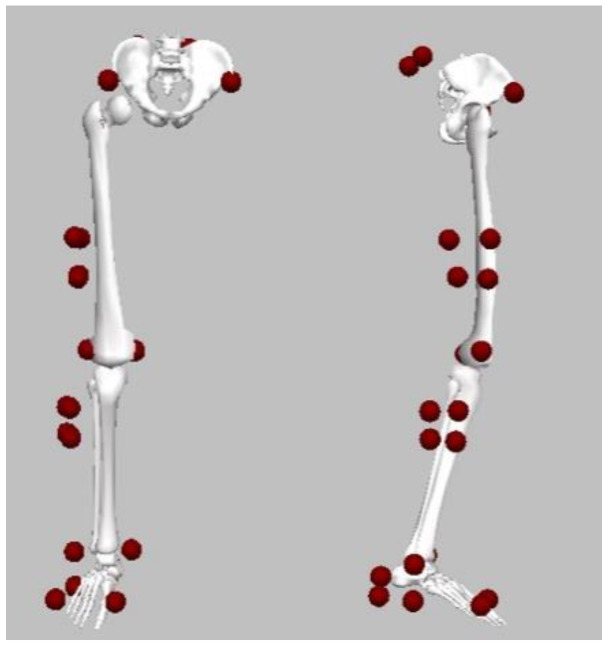
Illustration of the marker set.

After anthropometrical measurements taken, twenty-one reflective markers (ø = 14 mm) were attached over the pelvis (left and right ASIS and PSIS) and the right lower-limb on the medial and lateral femoral epicondyles, the medial and lateral malleoli and the first and fifth metatarsal heads. Four markers on rigid clusters were attached to the right shank and thigh, and an additional three markers on the superior, inferior and lateral heel counter were used to track the movement trials (Figure 2) [6]. The markers on the malleoli and epicondyles were used in static trial but removed in dynamic cutting trials. To keep consistency across conditions, the same researcher performed marker placements and provided verbal instructions.

After a 10-min warm-up, including self-selected stretching, jogging and familiarization with each of the tested maneuvers, the participants were instructed to tighten their shoelaces as they would in regular training. They were required to perform the tasks as fast as possible, plant their right foot on the force platform and change direction to the respective endpoints (Figure 1b). To ensure maximum-effort trials, three successful trials were collected for each condition, which is adequate for obtaining reliable biomechanics in sport cutting tasks [34]. A trial was considered valid if the right foot only fully contacted the force platform. A trial was discarded if an obvious slip and loss of balance was observed. The order of the cutting tasks and shoe conditions was randomized using an online program (http://www.random.org, assessed at 10 October 2019) across participants.

### 2.5. Data Processing

A spline interpolation was performed for missing marker trajectories using three frames of data before and after the missing data [35]. Data were further processed in Visual3D software (C-Motion Inc., Germantown, Philadelphia, PA, USA). Marker trajectories and ground reaction forces were filtered with the same fourth-order Butterworth filter with a cut-off frequency of 12 Hz to avoid artefacts being induced in inverse dynamics calculations [36]. For the ground reaction force parameters, the analogue signals were filtered with a fourth-order Butterworth filter with a 50 Hz cut-off frequency.

Contact time between initial foot contact to toe-off was defined using a 20N threshold of the vertical ground reaction force. The resultant horizontal ground reaction force was calculated by the square root of the sum of the squared antero-posterior and medial-lateral grounds reaction forces. Sagittal and coronal plane ankle kinematics angles, velocities and moments were assessed where the sequence of rotations (+/−) was along the medio-lateral X-axis (flexion/extension) and antero-posterior Y-axis (ankle inversion/eversion). Inverse dynamics was used to calculate the internal joint moments (i.e., applied by the muscles) using the resultant approach. Ankle moments were resolved into the proximal segment (shank) coordinate system using Dempster’s inertial parameters [37].

### 2.6. Statistical Analysis

The group mean and standard deviation were calculated from the three trials for statistical analyses. For all kinematic and kinetic parameters in each participant and shoe condition, the mean time-normalized curves were computed to 101 data points during the ground contact phase. Most cutting-related studies investigated discrete variables that were zero-dimensional (0D) [5,6,12,28,33,38], despite the biomechanical data collected being one-dimensional (1D). This results in bias in the 0D results because this data type should be modeled with different randomness [39]. We applied statistical parametric mapping (SPM) to remove the limitations associated with selection of discrete parameters for time-series data because we were not certain during which phases of the change of direction maneuvers the later upper support would be effective [40]. The SPM analysis was conducted in MatLab (R2020a, 9.8.0, The Mathworks Inc., Natick, MA, USA). The source code was downloaded from https://spm1d.org/Downloads.html (accessed on 2 April 2022) and the SPM repeated measures ANOVA test was applied to compare differences between the shoe conditions. Significance was determined by the SPM(F) value exceeding the critical threshold, which is calculated by random field theory on equivalent random smooth data [40]. Significant ANOVA results were followed-up with post-hoc SPM paired *t*-tests between all shoe conditions. The alpha level for all tests was 0.05, and no adjustments were made for the multiple comparisons because this study was exploratory in nature. The null hypothesis was that there would be no differences in the kinetic and kinematic parameters tested during the change of the direction step. 

The contact phase of cuts has commonly been divided into the braking and propulsion phases [6,11,27,31]. Based on these studies, which defined the braking and propulsion phases with maximum knee flexion angle, we descriptively interpreted the braking phase (0–40%) and propulsion phase (41–100%) of all cutting maneuvers in the discussion to enable comparisons with past results.

## 3. Results

### 3.1. Ground Reaction Forces

**Figure 3:**
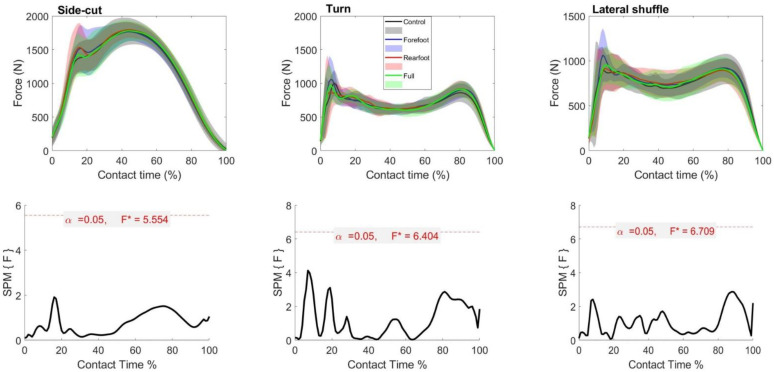
Mean (SD) vertical ground reaction force results across participants for each shoe condition (Black = Control, Blue = Forefoot, Red = Rearfoot; Green = Full) (**top**). The SPM(F) values across ground contact time (**bottom**). F* = critical value.

**Figure 4:**
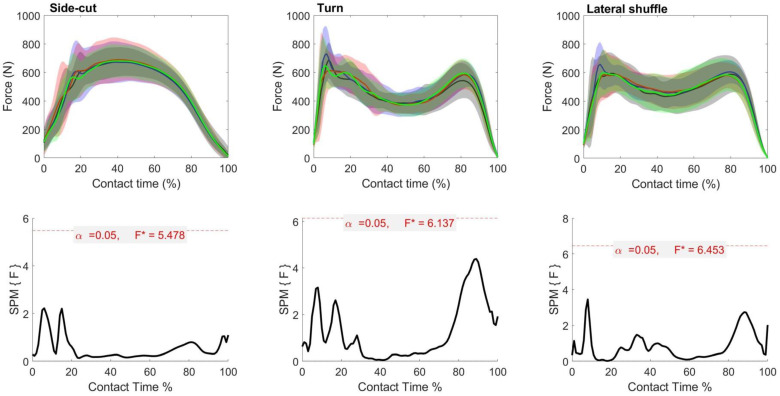
Mean (SD) resultant horizontal ground reaction force results across participants for each Scheme. F* = critical value.

The SPM repeated measures ANOVA revealed no differences in the vertical ground reaction force or resultant horizontal shear force during the change of direction step in any of the cutting maneuvers (Figure 3 and Figure 4). 

### 3.2. Ankle Kinematics

**Figure 5:**
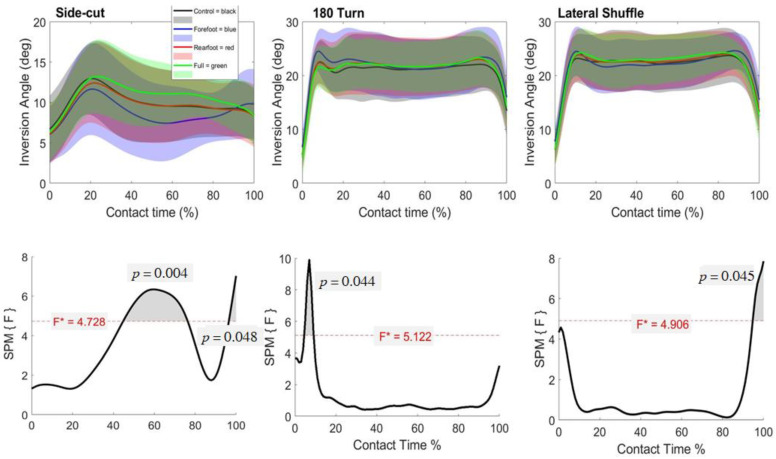
Mean (SD) frontal ankle angle across participants for each shoe condition (Black = Control, Blue = Forefoot, Red = Rearfoot; Green = Full) (**top**). The SPM(F) values across ground contact time (**bottom**). F* = critical value.

The SPM repeated measures ANOVA revealed no differences in the sagittal ankle angle during any of the cutting maneuvers (*p* > 0.05). There was a main effect in the frontal plane ankle angle in side-cut, turn and lateral shuffle (Figure 5). In the side cut, post-hoc analysis revealed significantly reduced inversion in the forefoot support compared to the full-length support shoes in between 44–80% of ground contact. Opposing this, there was increased inversion in the rearfoot support compared to forefoot support shoes between 98–100% of ground contact. In the turn, post-hoc analysis revealed significantly increased inversion in the forefoot support compared to the control and rearfoot support during 7–9% and 5–9% of ground contact time, respectively. In the lateral shuffle, post-hoc analysis revealed significantly increased inversion in forefoot support compared to rearfoot and full-length support between 98–100% and 96–100% of ground contact time, respectively.

**Figure 6:**
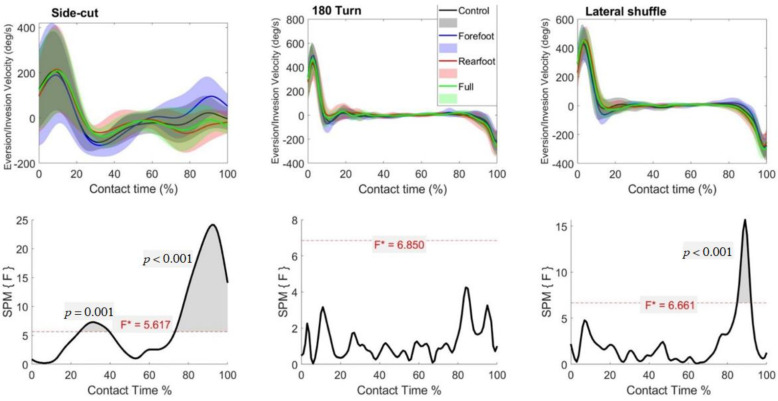
Mean (SD) frontal ankle velocity across participants for each shoe condition (Black = Control, Blue = Forefoot, Red = Rearfoot; Green = Full) (**top**). The SPM(F) values across ground contact time (**bottom**). F* = critical value.

There was a main effect in the frontal plane ankle velocity in the side-cut and lateral shuffle, but not the turn (Figure 6). In the side cut, post-hoc analysis revealed forefoot support shoes have increased inversion velocity during the latter stages of ground contact compared to the control (92–97%), rearfoot support (78–92%) and full-length support (77–100%) condition. Additionally, forefoot support had significantly increased eversion velocity than rearfoot support between 28–39% of contact time. The control also significantly increased inversion velocity compared to rearfoot support between 78–92% of ground contact. In the lateral shuffle, post-hoc analysis indicated forefoot support shoe significantly reduced the eversion velocity during the latter stages of the ground contact period compared to the rearfoot support (87–93%) and full-length support (88–93%) shoes.

### 3.3. Ankle Moments

**Figure 7:**
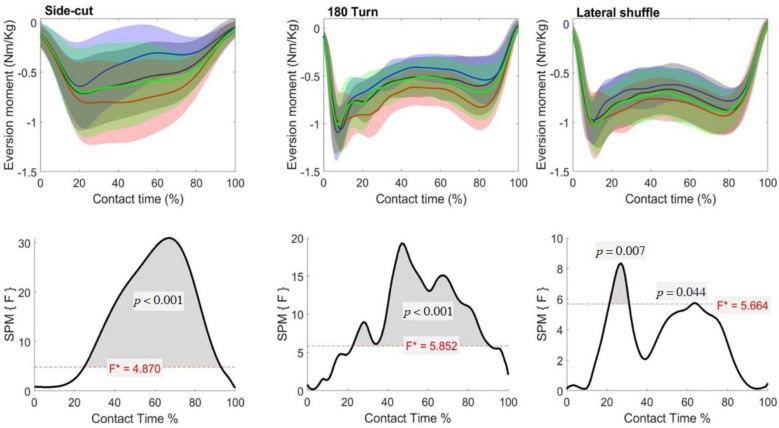
Mean (SD) frontal ankle moment across participants for each shoe condition (Black = Control, Blue = Forefoot, Red = Rearfoot; Green = Full) (**top**). The SPM(F) values across ground contact time (**bottom**). F* = critical value.

The SPM repeated measures ANOVA revealed no differences in the sagittal ankle moment during any of the cutting maneuvers. There was a main effect in the frontal plane ankle moment in the side cut, turn and lateral shuffle (Figure 7). In the side cut, post-hoc analysis revealed forefoot support shoes had a significantly reduced eversion moment compared to the control, rearfoot support and full-length support shoes between 34–78%, 22–91% and 42–95% of ground contact time, respectively. In addition, the control and full-length support shoes had a significantly reduced eversion moment compared to the rearfoot support shoes, with between 33–81% and 33–73% of ground contact, respectively.

In the turn, the post-hoc analysis also indicated a significantly reduced eversion moment in forefoot support compared to the rearfoot (23–88%) and full-length support shoes (44–48% and 94–97%). In addition, the control had a significantly reduced eversion moment compared to the rearfoot support shoe between 81–85% of ground contact. In the lateral shuffle, post-hoc analysis revealed a significantly reduced eversion moment in forefoot support shoes compared to rearfoot support shoes between 20–38% and 41–75% of contact time. 

## 4. Discussion

This study examined the influence of lateral shoe upper support (full-length, forefoot and rearfoot) on the ground reaction forces, and ankle kinematics and moments during side-cuts, complete turns and lateral shuffle maneuvers. The SPM results from the present study provided insights across the entire change of direction step, which enabled us to assess the effects of lateral upper support locations without any bias about what time points might provide differences. This was suitable for this exploratory analysis, as no prior data on lateral supports suggested we should limit our study to certain time points [40,41]. 

The present results indicated that both vertical and horizontal shear ground reaction forces are robust to different lateral shoe upper support constructions in all of the tested maneuvers. This suggests that the lateral upper support had a little to minimal effect on the ground reaction forces, which have been reported to be affected by other shoe construction features, such as midsole hardness and shear-reduction structure [11,31,42].

While sagittal plane motion appears to have an influence on ankle stability and inversion sprains, they were not influenced by the shoe lateral upper support in the present study. Yet, we found that the upper support structure played a role in the changes in coronal ankle variables including the inversion angle, inversion velocity and eversion moment in most of the change of direction maneuvers. The reduced ankle inversion angle and velocity during the braking phase of change-of-direction movements was suggested to reduce the risk of ankle sprain injury [12,43]. The forefoot support shoes increased ankle inversion between 8 and 14% of the turn compared with the control and full-length support shoes; the forefoot support shoes also increased ankle inversion in the propulsion phase (~96–100%) of the side-cut and lateral shuffle more than the rearfoot and full-length support shoes. Furthermore, the forefoot upper support shoes have increased inversion velocity during the propulsion phase of ground contact compared to control (92–96%), rearfoot (78–96%) and full (78–100%) upper support shoes. Larger inversion angle during early contact may link to increased ankle injury risk in forefoot support shoes during the complete turn, but this was not the case in the side-cut or lateral shuffle. 

On the other hand, the larger inversion velocity found in later contact phase may also allude to performance benefits, as it could promote better/faster support for inversion to push-off. It is appeared that the forefoot support shoes were increasing from middle (weight acceptance) to later phase (acceleration/push-off) of ground contact. This is explained by a coping effect between forefoot and midfoot/ankle. The previous studies have shown that medially or laterally lifting the forefoot through orthotics can cause subject-specific changes with a lateral shift of the center-of-pressure under the forefoot and, consequently, affect the loading of the knee joint in running [44,45]. Regarding joint kinetics, forefoot support shoes consistently showed the smallest eversion moment in either middle or late period in all of the tested movements, which indicated an effective stabilization of the ankle joint during cutting or turning. Such stabilization may reduce the fatigue of the muscle of the lower leg [46], thereby reducing ankle injury risk. However, further studies should investigate the interplay of segmental joint mobility, movement and stabilization provided by the extrinsic and intrinsic musculature that is required to coordinate and execute the high-demanding and complex kinematic cutting movements in forefoot-midfoot-tibia coupling.

Several efforts, such as shoe collars, heel counters, taping and foot inserts, have been developed to restrict large amounts of ankle inversion during cutting and turning tasks, which may be considered important for basketball ankle sprain injury prevention [8,10,43]. When compared to the control (i.e., no support), forefoot support could lead to a higher inversion angle during the early braking phase (~8–14%, turn) and inversion velocity in late propulsion phase (~92–96%, side cut) in the present study. On the other hand, shoes with rearfoot support could have the potential to induce smaller inversion velocity at propulsion phase (~78–96%, lateral shuffle). This could explain why the shoes with full-length support (forefoot plus rearfoot) did not show any differences in each of the tested maneuvers. The results suggest the effect of shoe upper support on ankle injury risk may be specific to the interaction among the cutting tasks and participant movement strategies. Future research can focus on if the forefoot support does pose an increased injury risk, how comfort is affected and if the push-off performance is influenced by the greater ankle inversion angle.

There are some limitations when interpreting our results. First, trained male athletes were recruited in this study and our data may not be generalizable to other playing levels or gender. It is known that females might be exposed to increased injury risks than males; moreover, future studies should examine the relationship between landing posture and lateral upper support structures to understand the underlying mechanism associated with ankle inversion injuries in basketball. Furthermore, an in-shoe robotic device could be considered to simulate various boundary conditions for better prediction of the calcaneal movement and ligamentous loading at the ankle. Lastly, we did not measure the knee and hip mechanics as well as muscle activation. Restriction of foot and ankle motion may lead to some compensations of knee motion. A future study should investigate this to confirm this association.

## 5. Conclusions

The lateral upper support of basketball shoes affects the coronal ankle mechanics, but not for ground reaction forces during maximum-effort cutting maneuvers. The forefoot lateral upper support shoes reduced the eversion moment but increased ankle inversion angle/velocity. The rearfoot lateral upper support may reduce the eversion moment and inversion velocity during propulsion compared to no upper support. These differences were found from early loading to late propulsion during the change of direction step, but were dependent on the cutting maneuver. The findings from this study may guide recommendations for how basketball shoes can be selected to alter ankle mechanics, and thus, optimize the cutting-related performances and injury risks for basketball players. 

## Data Availability

The data can be obtained from the corresponding authors upon reasonable request.

## References

[1] Abdelkrim B.N., El Fazaa S., El A.J. (2007). Time-motion analysis and physiological data of elite under 19-year-old basketball players during competition. Br. J. Sports Med..

[2] Brauner T., Zwinzscher M., Sterzing T. (2012). Basketball footwear requirements are dependent on playing position. Footwear Sci..

[3] Cormery B., Marcil M., Bouvard M. (2008). Rule change incidence on physiological characteristics of elite basketball players: A 10-year-period investigation. Br. J. Sports Med..

[4] Matthew D., Delextrat A. (2009). Heart rate, blood lactate concentration, and time-motion analysis of female basketball players during competition. J. Sports Sci..

[5] Cong Y., Lam W.K., Cheung J.T.M., Zhang M. (2014). In-shoe plantar tri-axial stress profiles during maximum-effort cutting maneuvers. J. Biomech..

[6] Lam W.K., Park E.J., Lee K.K., Cheung J.T. (2015). Shoe collar height effect on athletic performance, ankle joint kinematics and kinetics during unanticipated maximum-effort side-cutting performance. J. Sports Sci..

[7] De Luca J.F., Adams B.B., Yosipovitch G. (2012). Skin manifestations of athletes competing in the summer Olympics: What a sports medicine physician should know. Sports Med..

[8] Fong D.T., Hong Y., Chan L.K., Yung P.S.H., Chan K.M. (2007). A systematic review on ankle injury and ankle sprain in sports. Sports Med..

[9] Yu B., Garrett W.E. (2007). Mechanisms of non-contact ACL injuries. Br. J. Sports Med..

[10] Lam W.K., Kan W.H., Chia J.S., Kong P.W. (2022). Effect of shoe modifications on biomechanical changes in basketball: A systematic review. Sports Biomech..

[11] Cong Y., Lam W.K. (2021). Effects of shear reduction shoes on joint loading, ground reaction force and free moment across different cutting angles. J. Sport Sci..

[12] Liu H., Wu Z., Lam W.K. (2017). Collar height and heel counter-stiffness for ankle stability and athletic performance in basketball. Res. Sports Med..

[13] Worobets J., Wannop J.W. (2015). Influence of basketball shoe mass, outsole traction, and forefoot bending stiffness on three athletic movements. Sports Biomech..

[14] Langley B., Cramp M., Morrison S.C. (2019). The influence of motion control, neutral, and cushioned running shoes on lower limb kinematics. J. Appl. Biomech..

[15] Frederick E.C., Wojcieszak C., Shishoo R. (2005). Textile use in sport shoes. Textiles in Sports.

[16] McPoil T.G. (2000). Athletic footwear: Design, performance and selection issues. J. Sci. Med. Sport.

[17] Harrison K., Feeney D., Pryhoda M.K., Dicharry J., Nelson N.M., Shelburne K.B., Davidson B.S. (2021). Alternative upper configurations during agility-based movements: Part 2, joint-level biomechanics. Footwear Sci..

[18] Pryhoda M.K., Wathen R.J., Dicharry J., Shelburne K.B., Feeney D., Harrison K., Davidson B.S. (2021). Alternative upper configurations during agility-based movements: Part 1, biomechanical performance. Footwear Sci..

[19] Subramanium A., Honert E.C., Cigoja S., Nigg B.M. (2021). The effects of shoe upper construction on mechanical ankle joint work during lateral shuffle movements. J. Sport Sci..

[20] Lam W.-K., Liebenberg J., Woo J., Park S.-K., Yoon S.-H., Cheung R.T., Ryu J. (2018). Do running speed and shoe cushioning influence impact loading and tibial shock in basketball players?. Peer J..

[21] Apps C., Rodrigues P., Isherwood J., Lake M. (2019). Footwear insoles with higher frictional properties enhance performance by reducing in-shoe sliding during rapid changes of direction. J. Sport Sci..

[22] Hosein R., Lord M. (2000). A study of in-shoe plantar shear in normals. Clin. Biomech..

[23] Lam W.K., Cheung C.C.W., Leung A.K.L. (2020). Shoe collar height and heel counter-stiffness for shoe cushioning and joint stability in landing. J. Sport Sci..

[24] Riemann B.L., Lephart S. (2002). Sensorimotor system 1: Physiological basis of joint stability. J. Athl. Train..

[25] Kinchington M., Ball K., Naughton G. (2012). Relation between lower limb comfort and performance in footballers. Phys. Ther. Sport.

[26] Schreurs M.J., Benjaminse A., Lemmink K.A. (2017). Sharper angle, higher risk? The effect of cutting angle on knee mechanics in invasion sport athletes. J. Biomech..

[27] Besier T.F., Lloyd D.G., Cochrane J.L., Ackland T.R. (2001). External loading of the knee joint during running and cutting maneuvers. Med. Sci. Sports Exerc..

[28] Havens K.L., Sigward S.M. (2015). Whole body mechanics differ among running and cutting maneuvers in skilled athletes. Gait Posture.

[29] Van Melick N., Meddeler B.M., Hoogeboom T.J., Nijhuis-van der Sanden M.W.G., van Cingel R.E.H. (2017). How to determine leg dominance: The agreement between self-reported and observed performance in healthy adults. PLoS ONE.

[30] Dos’Santos T., Thomas C., Comfort P., Jones P.A. (2018). The effect of angle and velocity on change of direction biomechanics: An angle-velocity trade-off. Sports Med..

[31] Lam W.K., Qu Y., Yang F., Cheung R.T.H. (2017). Do rotational shear-cushioning influence horizontal ground reaction forces and perceived comfort during basketball cutting maneuvers?. Peer J..

[32] Stojanovic E., Stojiljkovic N., Scanlan A.T., Dalbo V.J., Berkelmans D.M., Milanovic Z. (2017). The activity demands and physiological responses encountered during basketball match-play: A systematic review. Sports Med..

[33] Havens K.L., Sigward S.M. (2015). Joint and segmental mechanics differ between cutting maneuvers in skilled athletes. Gait Posture.

[34] Mok K.M., Bahr R., Krosshaug T. (2017). Reliability of lower limb biomechanics in two sport-specific sidestep cutting tasks. Sports Biomech..

[35] Sigward S.M., Powers C.M. (2007). Loading characteristics of females exhibiting excessive valgus moments during cutting. Clin. Biomech..

[36] Kristianslund E., Krosshaug T., van den Bogert A.J. (2012). Effect of low pass filtering on joint moments from inverse dynamics: Implications for injury prevention. J. Biomech..

[37] Dempster W.T. (1955). Space requirements of the seated operator: Geometrical, kinematic, and mechanical aspects of the body, with special reference to the limbs. WADC Technical Report.

[38] Havens K.L., Sigward S.M. (2015). Cutting mechanics: Relation to performance and anterior cruciate ligament injury risk. Med. Sci. Sports Exerc..

[39] Pataky T.C., Vanrenterghem J., Robinson M.A. (2015). Zero- vs. one-dimensional parametric vs. non-parametric, and confidence interval vs. hypothesis testing procedures in one-dimensional biomechanical trajectory analysis. J. Biomech..

[40] Pataky T.C., Robinson M.A., Vanrenterghem J. (2013). Vector field statistical analysis of kinematic and force trajectories. J. Biomech..

[41] Vanrenterghem J., Venables E., Pataky T., Robinson M.A. (2012). The effect of running speed on knee mechanical loading in females during side cutting. J. Biomech..

[42] Leong H.F., Lam W.K., Ng W.X., Kong P.W. (2018). Center of pressure and perceived stability in basketball shoes with soft and hard midsoles. J. Appl. Biomech..

[43] Zhang S., Wortley M., Chen Q.J., Freeman J. (2009). Efficacy of an ankle brace with a subtalar locking system in inversion control in dynamic movements. J. Orthop. Sport Phys. Ther..

[44] Nigg B.M., Stergiou P., Cole G., Stefanyshyn D., Mundermann A., Humble N. (2003). Effect of shoe inserts on kinematics, center of pressure, and leg joint moments during running. Med. Sci. Sport Exerc..

[45] Zhang X., Lam W.K., Vanwanseele B. (2022). Dose-response effects of forefoot and arch orthotic components on the center of pressure trajectory during running in pronated feet. Gait Posture.

[46] Jorgensen U. (1990). Body load in heel-strike running: The erect of arm heel counter. Am. J. Sport Med..

